# Corticosteroid monotherapy versus combined cytarabine continuous rate infusion and corticosteroid therapy in dogs with meningoencephalitis of unknown origin: A blinded, randomized, controlled trial

**DOI:** 10.1111/jvim.17088

**Published:** 2024-05-03

**Authors:** Bethan S. Jones, Francois Xavier Liebel, Angela Fadda, Sophie Martin, Richard Lawn, Kali Lazzerini, Thomas Harcourt‐Brown

**Affiliations:** ^1^ Langford Vets Small Animal Hospital Bristol United Kingdom

**Keywords:** dog, meningoencephalitis of unknown origin, neurology, noninfectious meningoencephalitis

## Abstract

**Background:**

Treatment options available for meningoencephalitis of unknown origin (MUO) in dogs are suboptimal, and currently, no single treatment protocol appears to be superior.

**Objectives:**

Compare neurological deterioration rates at 7 days between dogs with MUO treated with corticosteroids alone or combined with cytosine arabinoside (CA) continuous rate infusion (CRI) and compare clinical deterioration and survival at 30 and 100 days.

**Animals:**

Sixty‐nine dogs with magnetic resonance imaging (MRI) and cerebrospinal fluid (CSF) features or both compatible with MUO.

**Methods:**

Parallel, blinded, randomized controlled trial. Simple randomization into 2 treatment groups: 4 mg/kg/day prednisolone (or dexamethasone equivalent) for 2 days or 200 mg/m^2^ CA CRI over 8 hours plus 2 mg/kg/day prednisolone. Blinding of the treatment protocol was carried out using reversible redaction of clinical records, and treatment failure was defined as deterioration of neurological assessment or death. Using intention‐to‐treat analysis, proportions failing treatment at 7, 30, and 100 days were compared using Fisher's exact test. All‐cause mortality at 100 days was compared using Kaplan‐Meier survival curves.

**Results:**

Thirty‐five dogs were allocated to corticosteroid only, and 34 dogs were allocated to combined CA CRI and corticosteroid. Proportions failing treatment at 7, 30, and 100 days were 7/35 (20%), 9/35 (26%), and 15/35 (43%) in the corticosteroid‐only group and 8/34 (24%), 11/34 (32%), and 23/34 (68%) in the corticosteroid and CA CRI group. All‐cause mortality at 100 days was not significantly different between groups (*P* = .62). Clinically relevant treatment‐related adverse effects were not observed.

**Conclusions and Clinical Importance:**

We found no difference in outcome between corticosteroid monotherapy and combined cytarabine CRI and corticosteroid therapy at 7, 30, and 100 days after diagnosis in dogs with MUO.

**Abbreviations**
CA: cytosine arabinoside
CRI: continuous rate infusion
CSF: cerebrospinal fluid
MRI: magnetic resonance imaging
MUO: meningoencephalitis of unknown origin
NME: necrotizing meningoencephalitis

## 1 INTRODUCTION

Meningoencephalitis of unknown origin (MUO) in dogs is a broad term for a group of inflammatory brain diseases that can only be differentiated histopathologically, including granulomatous meningoencephalitis and necrotizing meningoencephalitis (NME). The pathogenesis of MUO is still unclear, but it is hypothesized that an immune‐mediated process is involved based on the histological and cytological presence of inflammatory cells and autoantibodies in astrocytes.^1^, ^2^ Treatment of MUO has, therefore, been aimed at immunosuppression using corticosteroids and other immunosuppressive drugs such as cytosine arabinoside (CA), leflunomide, and cyclosporine.^3^, ^4^, ^5^ The best treatment protocol for achieving remission in dogs with meningoencephalitis is still unknown and the prognosis remains guarded.^6^, ^7^ Several case series suggest that a large proportion of dogs die or are euthanized in the first 7 to 100 days after diagnosis,^8^, ^9^ suggesting an increased at‐risk period of mortality immediately after diagnosis. A previous study reported a median survival time of 602 days for dogs that received immunosuppressive doses of corticosteroids, supporting the use of prednisolone monotherapy in the treatment of meningoencephalitis.^10^ A common protocol is the addition of CA to corticosteroids, and whereas 1 study found that treatment with CA continuous rate infusion (CRI) at the time of diagnosis improved 3‐month survival times compared with those dogs that received SC injection of CA,^5^ and another retrospective study showed that the addition of CA to cyclosporine and prednisone at the time of diagnosis did not improve outcomes in dogs with MUO.^11^ However, regardless of treatment choice, the survival rate is variable, and a proportion of dogs can continue to deteriorate despite treatment. Several studies have identified risk factors for deterioration or negative prognostic indicators such as the presence of seizures, multifocal or brainstem lesions, and certain magnetic resonance imaging (MRI) features including evidence of cystic lesions that are T1W hypointense associated with necrosis and more suggestive of a necrotizing encephalitis.^7^, ^12^, ^13^, ^14^ A more recent study identified that the presence of obtundation was the highest risk factor for early euthanasia.^9^

To date, no randomized prospective trials have compared treatment of meningoencephalitis with corticosteroid monotherapy and combined corticosteroid and CA CRI. Our primary objective was to compare the outcome at 7‐days post‐diagnosis in dogs treated with immunosuppression using corticosteroids alone to dogs treated with corticosteroids and CRI of CA.

Our secondary aims were to compare outcomes at 30 and 100 days between treatment groups and describe complicating factors such as owner compliance and alterations in the medication protocol by referring veterinarians.

## 2 METHODS

### 2.1 Trial design

We performed a randomized, blinded, parallel‐group controlled trial.

Ethical approval for the prospective study was obtained from the University ethics board (VIN/18/052).

A power calculation was performed with previous data^5^, ^13^ using WebPlotDigitizer (Copyright 2010‐2021 Ankit Rohatgi [automeris.io]). The number of animals needed per group was 33 dogs with a power of 80% at a level of significance of .05 to detect a 30% difference in survival.

### 2.2 Case selection

Dogs presented to Langford Veterinary Services, Bristol, United Kingdom, between July 2019 and July 2022 with suspected MUO were actively recruited into the study. Data collected included signalment, history including duration of clinical signs before presentation, and general physical and neurological examination findings. Inclusion criteria were based on guidelines from a meta‐analysis on noninfectious meningoencephalitis^15^: (1) dogs > 6 months of age with neurological examination findings and neuroanatomical localization consistent with inflammatory brain disease, (2) presence of an inflammatory response on cerebrospinal fluid (CSF) analysis (mononuclear or lymphocytic pleocytosis with total nucleated cell count [TNCC] >5 cells/μL and >50% mononuclear cells), (3) MRI results compatible with non‐infectious, inflammatory etiology, and (4) negative serological testing for the geographical area (*Toxoplasma gondii* and *Neospora caninum*).

Dogs were excluded if they had received any immunosuppressants before presentation, they tested positive for infectious causes (Toxoplasma and Neospora) or if their follow‐up was incomplete.

Dogs were still included if they had (1) abnormal MRI findings compatible with inflammatory disease but normal CSF analysis, (2) normal MRI findings with abnormal CSF cytology or (3) abnormal MRI findings but CSF sampling was not attempted because of concerns for increased intracranial pressure.

Magnetic resonance imaging was performed using a 1.5 Tesla scanner (Phillips Ingenia); sequences varied but all of the cases included T2‐weighted sagittal and transverse, T1‐weighted transverse, T2‐fluid‐attenuating inversion recovery, and postcontrast transverse T1‐weighted images of the brain at minimum. Cases had CSF analysis (where possible), serology and polymerase chain reaction (PCR) for infectious diseases (Toxoplasma and Neospora), and serum creatine kinase (CK) and aspartate aminotransferase (AST) activities measured pending infectious disease results. Serum CK and AST activities were used to guide infectious disease testing before immunosuppressive treatment based on a previously published study,^16^ allowing for prompt treatment of all cases at a similar time interval after investigations.

### 2.3 Interventions and randomization

After investigations contributing to high clinical suspicion of MUO, consent for enrollment in the study was obtained by the clinician in charge of the case, and the author (Bethan Jones) was contacted for randomization. Cases were randomized into 1 of 2 treatment groups using a simple randomization smartphone app (PickOne Theta Labs Inc.):

- Group 1—dexamethasone monotherapy 0.6 mg/kg (equivalent to 4 mg/kg prednisolone) IV once daily for 2 days
- Group 2—combined lower dose of dexamethasone 0.3 mg/kg IV (equivalent to 2 mg/kg prednisolone) once daily for 2 days and 200 mg/m^2^ CA CRI over 8 hours.

A higher initial dose of corticosteroid in the corticosteroid‐only treatment group was chosen to allow for the option of rescue therapy for dogs failing treatment, so that dogs randomized to the cytarabine group could receive an additional 0.3 mg/kg dexamethasone (or prednisolone equivalent). Therefore, dogs failing treatment in either treatment group could receive the other treatment.

**FIGURE 1:**
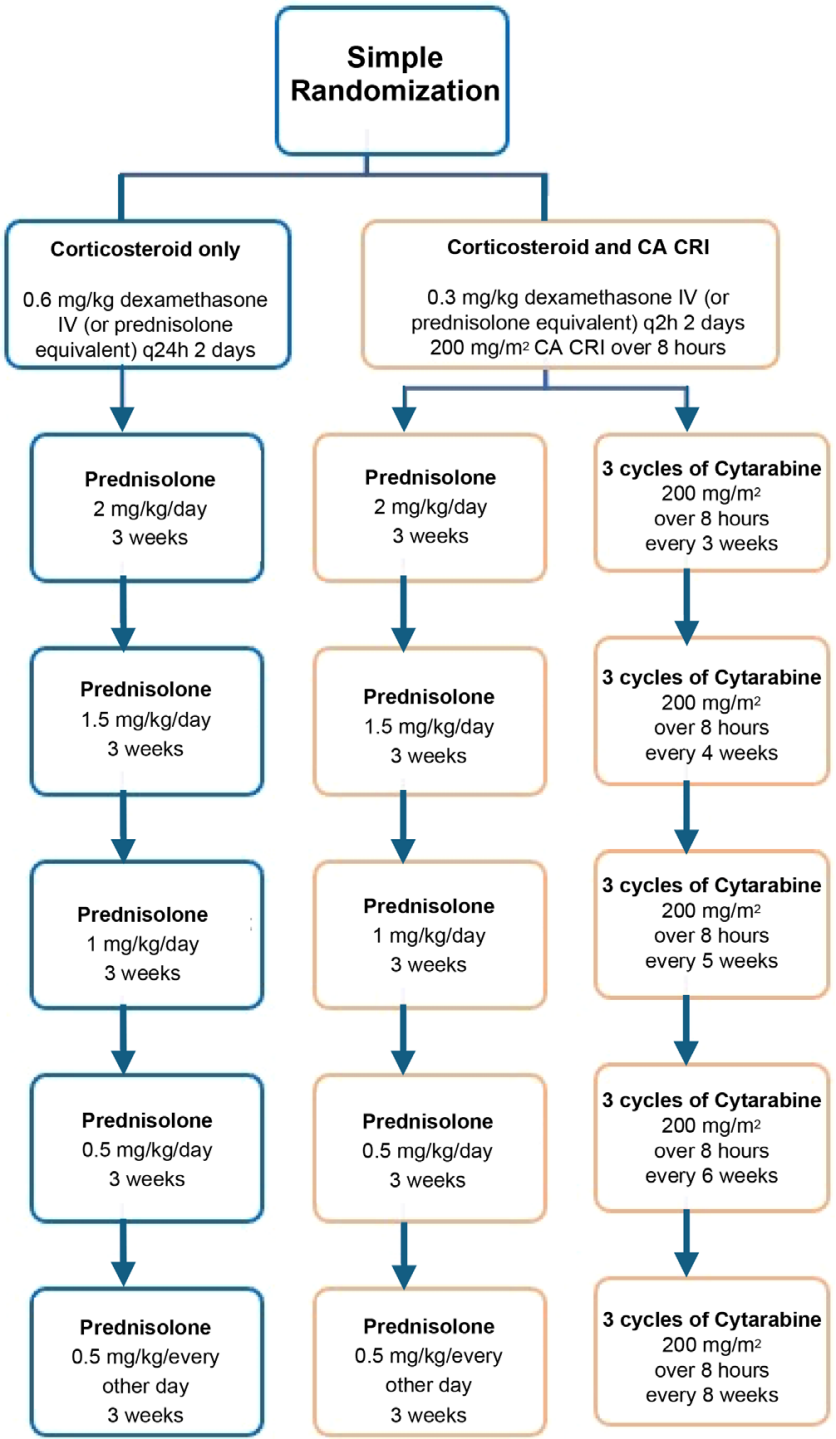
Treatment protocols following case identification and randomization.

Both groups subsequently received a tapering PO prednisolone course over 3 months starting at 2 mg/kg/day PO according to normal practice protocol (outlined in Figure 1). Dogs that received the initial CA CRI could continue with repeat infusions according to the normal practice protocol.

### 2.4 Neurological scoring of dogs and blinding of treatment allocation

A neurological scoring system was developed by authors Bethan Jones and Tom Harcourt‐Brown (see Supporting Information S1). A full neurological examination was performed and recorded at the time of admission by a neurology resident‐in‐training or by a board‐certified neurologist, whereas the author (Bethan Jones), owner, and clinician in charge of the case were aware of the allocated treatment group, and outcome assessors were kept blinded to the allocation.

Dogs were hospitalized for a minimum of 24 to 48 hours before being discharged to allow for scoring of their neurological deficits to evaluate for signs of deterioration. Scoring was performed by the same European board‐certified neurologist, or by a neurology resident‐in‐training, who was blinded to the treatment type received. Scoring took place 12 to 24 hours after commencement of treatment and continued twice daily until discharge from the hospital. To assure blinding, dogs in both groups were handled with appropriate personal protective equipment as if they had received cytotoxic drugs. Hospital records were partially covered to prevent the unblinding of the clinician scoring the dogs but, at the same time, maintain health and safety.

Blinding was removed at the time of clinical deterioration or after 7 days, and dogs that deteriorated could receive the other treatment arm.

### 2.5 Outcome measures

The primary outcome of treatment failure was defined as death or deterioration based on a worsening neurological score at the same localization or displaying signs of a new localization up to 7 days after starting treatment. At this point, dogs that were deteriorating were allowed to cross over to the alternative protocol to allow the opportunity to have both arms of the treatment trial. After the 7 days, treatment allocation was unblinded, and treatment failure rates were compared at 30 and 100 days after diagnosis using an intention‐to‐treat analysis.

Secondary outcome measures were all‐cause mortality at 100 days using Kaplan‐Meier survival curves. Referring veterinary practice or owner contact was performed at 1 week, 1 month, and 3 months after discharge to obtain data including owner compliance and any drug‐related adverse effects observed.

Necropsies were performed to obtain a histopathological diagnosis where possible.

### 2.6 Statistical analysis

Data were analyzed using the commercially available statistical program GraphPad© and examined for normality by visual assessment of a quantile plot and the Shapiro‐Wilk test. Putative prognostic factors were compared between groups to ensure there was not a failure of randomization. Age (months) at presentation, duration (days) of clinical signs before referral, and CSF TNCC and total protein concentration were not normally distributed and descriptive statistics were expressed as median (range) and compared using the Mann‐Whitney *U* test. The proportion of dogs presenting with obtundation was compared using Fisher's exact test.

Based on an intention‐to‐treat analysis, the primary outcome of treatment failure defined as death or deterioration at 7, 30, and 100 days was compared between the 2 treatment groups using Fisher's‐exact test.

Secondary outcomes of all‐cause mortality at 100 days were compared using log‐rank analysis of Kaplan‐Meier survival curves.

A *P* value of <.05 was considered significant.

## 3 RESULTS

**FIGURE 2:**
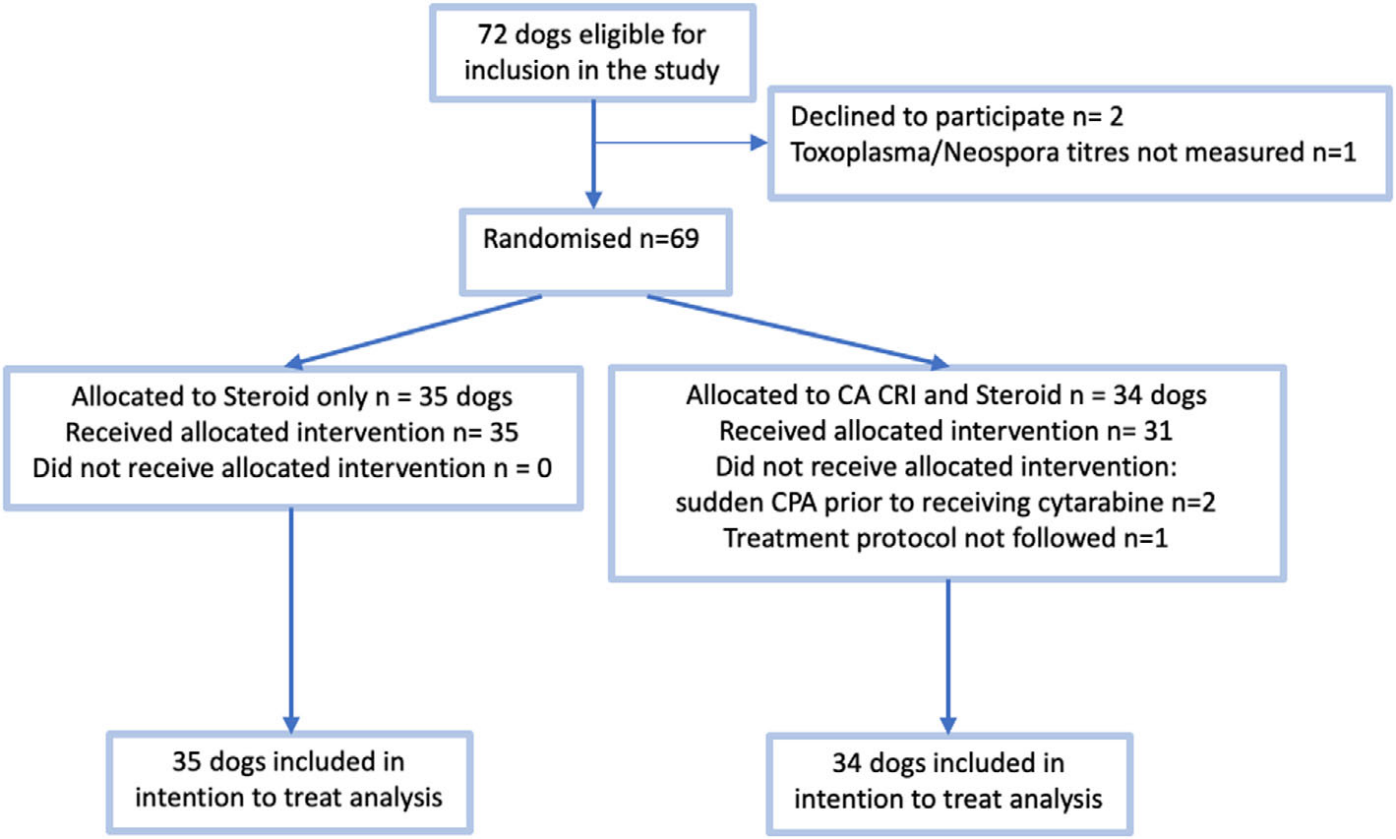
Numbers of dogs who were randomly assigned, received the intended treatment, and were analyzed for the primary outcome in each group.

Sixty‐nine dogs were identified and recruited into the study. After randomization, 35 dogs were in the corticosteroid‐only group and 34 dogs in the combined CA and corticosteroid group (Figure 2).

The most common breeds included French Bulldogs (12), Pugs (12), crossbreeds (11), Chihuahua (6), and West Highland White Terriers (6). The median age at presentation was 48 months (range, 9‐120 months). Thirty‐seven of 69 (54%) dogs were female (7 female intact and 30 female neutered) and 32/69 (46%) dogs were male (27 male neutered and 5 male intact). Clinical signs were described as acute onset and progressive in all cases, with the duration of signs ranging from 24 hours to 4 weeks before presentation.

Table 1 shows the distribution of age, duration of clinical signs and CSF analysis between the 2 treatment groups with no significant differences found between groups.

**TABLE 1:** Quantitative data for dogs in both treatment groups.

Quantitative Data collected	Corticosteroid only	Combined corticosteroid and CA CRI	*P* value Mann‐Whitney *U*	*P* value Fisher's exact test
Age months, median (range)	48 (9‐144)	60 (9–144)	.29	‐
Duration of clinical signs days, median (range)	3 (1‐30)	5 (1‐31)	.35	‐
CSF analysis				
Total nucleated cell count (TNCC) (cells/μL) median (range)	25.5 cells/μL (1‐4230)	46 cells/μL (3‐1183)	.74	‐
Total protein (mg/dL) median (range)	34.3 mg/dL (17‐495.7)	80 mg/dL (21.2‐214.1)	.17	‐
Number of dogs obtunded on presentation	11	10	‐	1

Twenty‐one dogs (30%) presented with obtunded mentation on initial neurological examination. Eleven of 35 (31%) dogs presented with obtundation in the corticosteroid‐only group and 10/34 (29%) dogs in the combined CA and corticosteroid group.

Cerebrospinal fluid analysis median TNCC was 209 cells/μL (range, 0‐4230). Cerebrospinal fluid was considered normal (<5 cells/μL) in 13 cases, but these had multifocal lesions on MRI compatible with an inflammatory process. Median total protein concentration was 79.04 mg/dL (range, 17.1‐495.7 mg/dL). Cerebrospinal fluid was not performed in 8 cases (12%) because of concern for increased intracranial pressure, 5/8 (62.5%) dogs in the combined CA and corticosteroid group, and 3/8 (37.5%) dogs in the corticosteroid‐only group. No cases had a normal MRI with abnormal CSF analysis.

### 3.1 Seven‐day outcome

**FIGURE 3:**
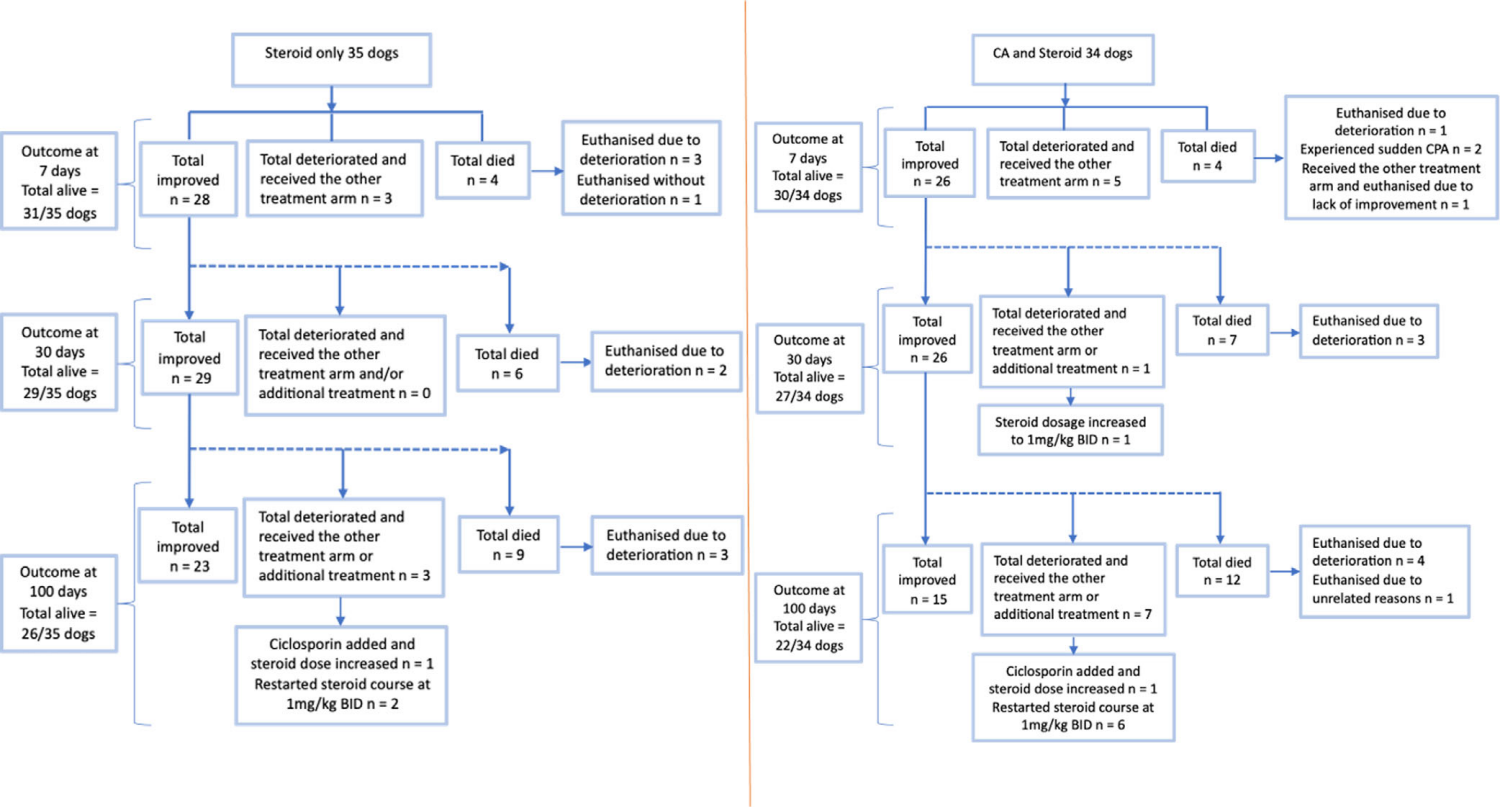
Flowchart documenting the numbers of dogs randomly allocated to each treatment group and following their outcome at 7, 30, and 100 days.

Figure 3 shows the proportions of dogs included in an intention‐to‐treat analysis and the primary outcome of treatment failure at 7, 30, and 100 days post‐treatment.

The proportion of dogs that died or deteriorated in the combined CA and corticosteroid group within the first 7 days was 8/34 dogs (24%) compared with 7/35 dogs (20%) in the corticosteroid‐only group (*P* = .78).

The mortality rate for the corticosteroid‐only group was 4/35 (11%) dogs within the first 7 days compared with 4/34 (12%) dogs in the combined CA and corticosteroid group. Two dogs died before receiving CA because of cardiac arrest, and 1 dog in the corticosteroid‐only group was euthanized 24 hours after receiving corticosteroid at the owner's request.

Eight of 69 dogs (12%) that failed the initial treatment protocol because of deterioration received the other treatment arm. Three of 8 cases were in the initial corticosteroid‐only group, and 5/8 cases were in the combined CA and corticosteroid group. Of these cases that received the other treatment arm within the first 7 days, 3/3 dogs in the initial corticosteroid‐only group that subsequently received CA CRI improved neurologically, and 4/5 dogs in the initial CA CRI and corticosteroid group that received additional corticosteroid improved. The remaining dog in the CA CRI and corticosteroid group that received additional corticosteroid continued to deteriorate and was euthanized 3 days after the initiation of treatment.

Necropsy was performed in 1 dog that died in the corticosteroid‐only group with findings compatible with lymphoplasmacytic MUO.

### 3.2 Thirty‐day outcome

Six dogs died or deteriorated within 8 to 30 days after the treatment start date, 2/6 dogs in the corticosteroid‐only group and 4/6 dogs in the CA and corticosteroid group. Only 1 of the 4 dogs in the combined CA and corticosteroid group continued with CA infusions after the first initial infusion and the corticosteroid dosage was increased. The remaining dogs were euthanized at the referring veterinary practice because of continued deterioration.

The overall proportion of dogs that died or deteriorated by 30 days within the combined CA and corticosteroid group was 11/34 dogs (32%) compared with 9/35 dogs (26%) in the corticosteroid‐only group (*P* = .60).

The all‐cause mortality rate at 30 days was 6/35 (17%) dogs in the corticosteroid‐only group and 7/34 (21%) dogs in the CA and corticosteroid group (*P* = .78).

### 3.3 Hundred‐day outcome

Eighteen dogs died or deteriorated within 31 to 100 days after the treatment start date, 6 dogs in the corticosteroid‐only group and 12 dogs in the CA and corticosteroid group.

The overall proportion of dogs that died or deteriorated within 100 days in the combined CA and corticosteroid group within 100 days was 22/34 dogs (65%) compared to 15/35 dogs (43%) in the corticosteroid‐only group (*P* = .09).

The mortality rate for the corticosteroid‐only group was 3/6 dogs and 5/12 dogs in the combined CA and corticosteroid group. One dog was euthanized because of corneal ulceration in the 1 remaining eye, requiring enucleation. Of the remaining 10/18 dogs that relapsed with neurological signs, 2 dogs (1 in the corticosteroid‐only group and 1 in the CA CRI and corticosteroid group) were started on cyclosporine in addition to increasing the corticosteroid dosage. The remainder of the dogs that deteriorated were restarted on the corticosteroid course at a dosage of 1 mg/kg prednisolone by the referring veterinarian.

**FIGURE 4:**
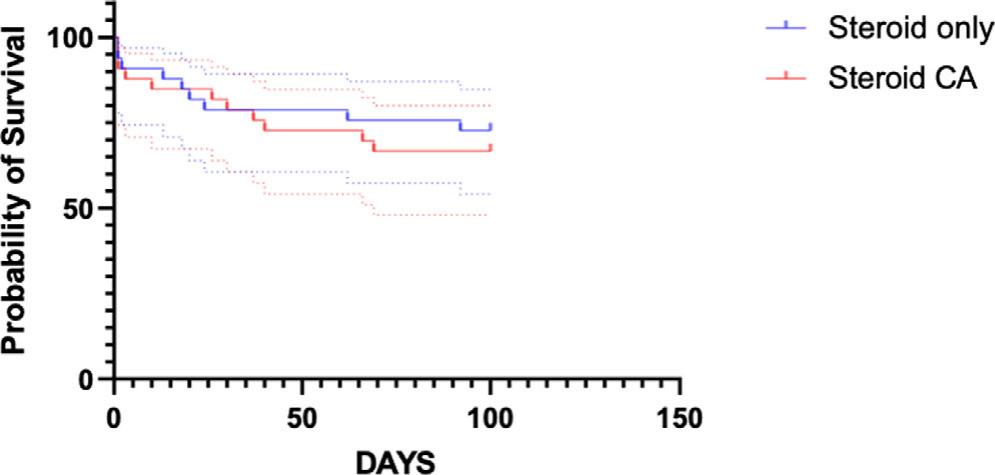
Survival at 100 days (all‐cause mortality) in dogs with meningoencephalitis of unknown origin treated with corticosteroids only or combined corticosteroid and cytosine arabinoside continuous rate infusion (CRI) at initial presentation.

Log‐rank analysis of the Kaplan‐Meier survival curves for the 2 treatment groups did not identify a significant difference in survival (log‐rank test, *P* = .62; Figure 4). The all‐cause mortality at 100 days was 9/35 (26%) dogs in the corticosteroid‐only group and 12/34 (35%) dogs in the CA and corticosteroid group (*P* = .44).

Five of 69 dogs (7%) had intra‐axial cystic lesions with T1W hypointensity, suggestive of necrosis suspected to be associated with NME. Three of 5 dogs were in the CA and corticosteroid group and 2/5 dogs were in the corticosteroid‐only group. All dogs improved and were alive at 100 days posttreatment.

After discharge, only 35% of cases (17/48) followed the full tapering corticosteroid plan set out in Figure 1. In many cases, referring veterinarians altered the dosage when neurological signs were suspected to be associated with relapse, and in some cases, corticosteroid usage was continued at a low dose (5 mg q24h or q48h) at the request of the owners because of fear of relapse. Twenty‐one dogs in the CA CRI and corticosteroid group only received 1 initial infusion of CA at diagnosis and did not continue for financial reasons or deterioration of their neurological signs. Only 7 dogs continued with CA infusions, with 2/7 dogs continuing infusions at the referring veterinary practice.

## 4 DISCUSSION

We performed a parallel, blinded, randomized controlled trial comparing corticosteroid monotherapy with combined CA CRI and corticosteroid therapy in the treatment of MUO in dogs. We did not find that the risk of death or deterioration was decreased with the addition of CA CRI to corticosteroid therapy when compared with initial treatment with corticosteroids alone within the first 7 days of treatment based on an intention‐to‐treat analysis. We chose to perform an intention‐to‐treat analysis to preserve randomization and remove the bias that a per‐protocol or as‐treated analysis would introduce into the study.^17^, ^18^

Survival analysis at 100 days did not show a significant difference between treatment groups. However, this analysis was more difficult to compare because of the inability to control treatment once patients had been discharged. The long‐term effect of CA in decreasing the relapse rate cannot be assessed because the majority of cases discontinued treatment after the first infusion and this aspect requires further investigation.

Our finding of a lack of improvement in outcomes in dogs with MUO is similar to findings reported in a recent retrospective study comparing corticosteroid‐only and a combined corticosteroid and CA CRI protocol^9^ and a retrospective study comparing the addition of CA (SC injection or CRI) at the time of diagnosis to cyclosporine and corticosteroid therapy.^11^ A possible explanation for the lack of significantly improved outcomes with additional CA CRI in our study is the limited pharmacological data regarding the dosage of this drug in dogs. An effective therapeutic dose of CA for the treatment of MUO is still unknown, and it is unclear whether a higher peak plasma concentration or prolonged duration at or above reported potential therapeutic concentrations within the CNS is more beneficial.^19^ However, the purpose of our study was to compare a common treatment protocol being used with 200 mg/m^2^ CA CRI over 8 hours.

Obtundation recently has been shown to be a significant prognostic indicator for survival in the first 100 days in dogs with MUO.^9^ We did not control for obtundation because of the presence of similar numbers of dogs presenting with obtundation on initial presentation in both treatment groups. Similarly, we did not exclude dogs that had MRI characteristics suggestive of necrotizing encephalitis because only a small percentage of dogs had evidence of cystic T1W hypointense lesions on MRI and similar proportions in each treatment group had this finding. Currently, no strong statistical evidence indicates that dogs with NME have a worse outcome, and a recent study has shown large overlap among the different phenotypes of MUO histopathologically.^20^

Histopathologic diagnosis was not achieved in the majority of cases, and as such, we may have inadvertently included dogs with neoplastic disease in our study. We do not perceive this factor to contribute substantial bias, because dogs with multifocal neoplasia were equally likely to be included in either treatment group. Repeat investigations were not performed in cases in which deterioration was suspected to represent a relapse of neurological signs relating to MUO, and as such additional structural CNS pathology or disease could not be ruled out.

We did not encounter any major adverse effects during the clinical trial with either treatment group, and both CA and corticosteroids were well tolerated. The main adverse effects reported by the owners were related to corticosteroid use, including polydipsia, polyuria, polyphagia, and skin changes. In the majority of cases, these were tolerated, except for 1 dog where the owner requested early tapering of the corticosteroid dosage. In cases where there was a concern for increased risk of gastric ulceration with high doses of corticosteroid, such as with brachycephalic breeds and their tendency to regurgitate, gastrointestinal protection was provided by prescribing omeprazole. Adverse gastrointestinal effects occurred in 1 dog, a French Bulldog, and the corticosteroid course was tapered more rapidly. Because of the intention‐to‐treat analysis, meaningful conclusions on drug‐related adverse effects cannot be drawn because factors such as the owner's wishes and financial concerns appear to have a strong impact on their occurrence.

The challenges faced during our clinical trial included maintaining the blinding of clinicians from the randomized treatment group and adherence to protocols during the treatment trial. Recruitment of cases into the study was not anticipated to be a concern because the treatment groups were not dissimilar to treatment protocols already being used. Our study was not double‐blinded because the main clinician in charge of the case was required to communicate the risks of the use of CA to the owner. This factor may have introduced some bias into the study; however, the neurological scoring system performed by the clinician blinded to the treatment group proved valuable to prevent early deviation from the treatment protocol to which the patient was randomized or euthanasia in dogs that may have otherwise improved. To improve study strength, blinding of treatment allocation could be continued in the data collection for the primary outcome using numerical, concealed, preassigned treatment groups to avoid any bias that may be introduced during data collection and analysis. The neurological scoring system used in our study has not yet been validated, but a similar neurodisability scoring system recently has been published in a small cohort of dogs with MUO.^21^

## 5 CONCLUSIONS

Our study found no difference in outcome with the addition of a CA CRI to corticosteroid therapy when compared to initial treatment with corticosteroids alone within the first 7 days of treatment. Survival analysis within the first 100 days posttreatment was not significantly different between treatment groups; however, additional randomized clinical trials are required to directly compare the long‐term effect of combined CA CRI and corticosteroids with corticosteroid monotherapy.

## CONFLICT OF INTEREST DECLARATION

Authors declare no conflict of interest.

## OFF‐LABEL ANTIMICROBIAL DECLARATION

Authors declare no off‐label use of antimicrobials.

## INSTITUTIONAL ANIMAL CARE AND USE COMMITTEE (IACUC) OR OTHER APPROVAL DECLARATION

Approved by the University of Bristol ethical committee VIN/18/052.

## HUMAN ETHICS APPROVAL DECLARATION

Authors declare human ethics approval was not needed for this study.

## Supporting information


**Data S1.** Supporting Information.
