# Supplementary figures and images for: Corticosteroid monotherapy versus combined cytarabine continuous rate infusion and corticosteroid therapy in dogs with meningoencephalitis of unknown origin: A blinded, randomized, controlled trial
Source: J Vet Intern Med. 2024 May 3;38(3):1618–25. doi: 10.1111/jvim.17088 (PMC11099798; doi:10.1111/jvim.17088)

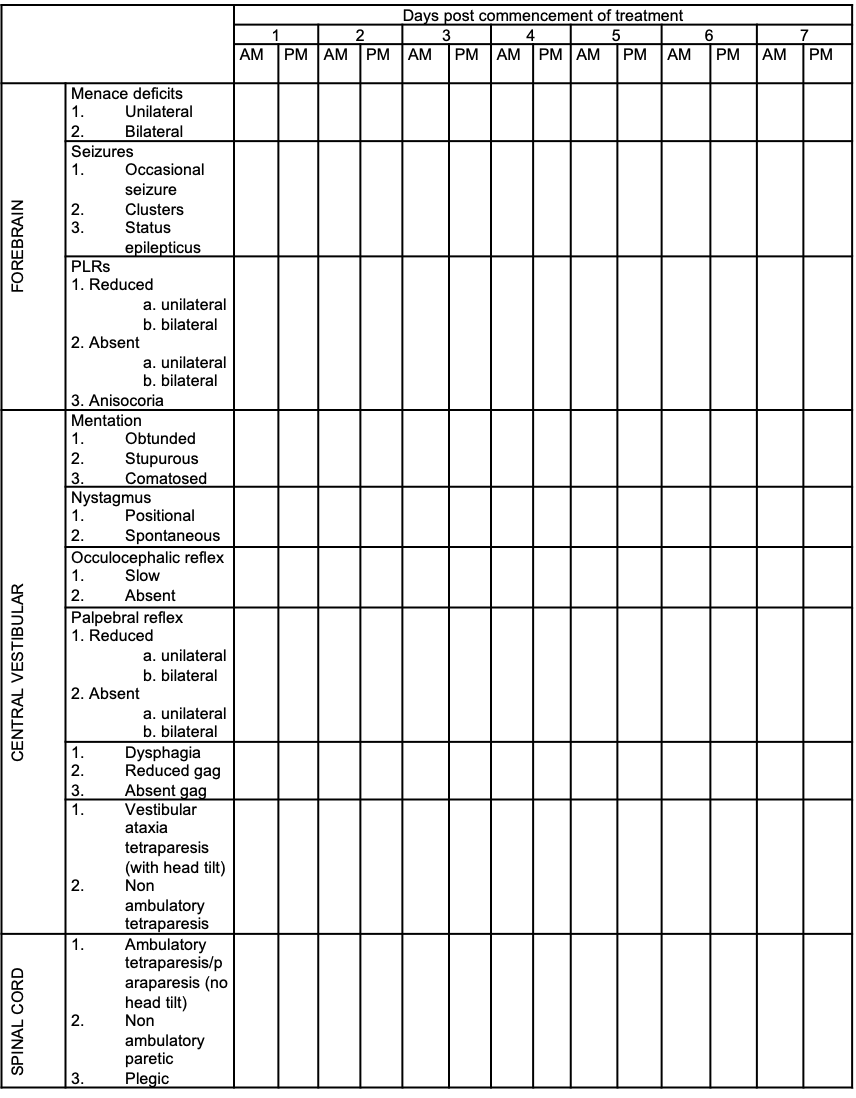


*Neurological scoring system.*

Supplement: Supplementary file 1 — Data S1. Supporting Information. [file JVIM-38-1618-s001.docx]
